# Medical screening of elderly Heads of State

**DOI:** 10.1177/01410768261447628

**Published:** 2026-05-10

**Authors:** Narinder Kapur, Shahina Begam

**Affiliations:** 1 University College London, London, UK; 2 Imperial College Healthcare NHS Trust, London, UK; 3 Imperial College Medical School, London, UK

## Abstract

As the world population grows older, and as work retirement ages are being increasingly abolished in many countries, it is more likely that there will be elderly Heads of State who hold powerful positions which can result in great good or great harm. On the basis of this narrative review, we propose that elderly Heads of State over the age of 70 years have annual medical screening that includes comprehensive neuropsychological testing, psychiatric screening and brain imaging, so that we can be reassured of the mental integrity of such leaders and so as to prevent harmful decisions being taken by mentally-compromised Heads of State.

In the last 100 years, there has been a transformation in the well-being of most citizens of the world, largely brought about by advances in medicine and technology. One of the key manifestations of this transformation has been that people are now living much longer. In addition, many countries have abolished work retirement age, such that many workers – in particular those in professional positions – are now in elderly age bands. In many countries, including the United States and the United Kingdom, there are no age limits for those who wish to hold positions in political or government organisations.

Gong et al.^1^ stated how cognitive deterioration of politicians is a critical emerging issue and proposed that cognitive assessments for politicians can be valuable for maintaining properly functioning governance. Gong et al. argued that cognitive assessments should be detailed, and not just cognitive screening tests, and that six key cognitive domains should be covered – learning and memory, executive function, complex attention, perceptual-motor function, language and social cognition. They further propose that the results of the cognitive assessment should be made public, and they acknowledge that some politicians may be reluctant to undergo cognitive assessment. Others have argued, such as Dr John Gartner and Professor Howard Gardner in their commentaries on US President Donald Trump, that since some Heads of State have access to nuclear buttons that could be used in a nuclear war, which could result in the end of civilisation, this provides even more imperative for their words and actions to be subject to scientific scrutiny. There have been recent articles published specifically in respect of Donald Trump,^2,3^ and a congresman has written to President Trump’s personal physician to ask that he undergo a medical examination that includes a comprehensive neuropsychological assessment.^4^

In his recent account of his career as physician to several US Presidents, Dr Jeffrey Kuhlman^5^ succinctly summarised relevant research, ‘*Research has shown that concept formation, abstraction, and mental flexibility all tend to decline with age, especially after age seventy. Older adults tend to think more concretely than younger adults. Aging also negatively impacts response inhibition, the ability to restrain an automatic response in favour of producing a novel response’*. Although Kuhlman cites the Mini Mental State Examination (MMSE) and the Montreal Cognitive Assessment (MOCA) as cognitive assessment tools, most neuropsychologists would argue that they are relatively crude instruments, often resulting in false negative findings, and having limitations in distinguishing between forms of dementia.^6^ Most neuropsychologists would therefore also agree with Gong et al.^1^ that a comprehensive cognitive assessment needs to be conducted so as to obtain an accurate picture of the state of mind of a political figure holding high office. Since such an assessment is more sensitive to disease processes that affect functioning of the brain, it is more likely to pick up signs of a neurodegenerative condition.

There has been debate as to whether Ronald Reagan, who died with Alzheimer’s disease, had elements of that disease during his time in office. Some have argued that this was evident in his press conferences,^7^ whereas others have argued that the evidence does not warrant such conclusions.^8^ Regardless, there has been an increasing interest in ageing Heads of State, and in particular whether cognitive decline or dementia may be present.^9^

In recent years, there have been major advances not only in neuropsychological assessment but also in brain imaging technologies and other biomarkers that help to detect the presence of neurodegenerative conditions.^10,11^ It is acknowledged that frontal lobe function specifically deteriorates with age in the normal population, and frontotemporal dementia may be misdiagnosed for years simply because the behaviours in question have been dismissed as just eccentric behaviour by the individual in question.

Recent developments, such as government decisions that adversely affect aid to low and middle income countries and federal researchers in the United States, have understandably given voice to concerns, especially – in the case of aid reduction – that this may result in the loss of thousands or even hundreds of thousands of lives.^12–15^ Those with direct experience of both politics and medicine have also highlighted forms of personality disorder such as hubris that may occur in Heads of State, and have outlined proposals similar to ours.^16–19^

We recommend that Heads of State over the age of 70 years have an annual medical assessment that includes both a comprehensive neuropsychological examination together with brain imaging and other biomarkers that detect the presence of cerebral pathology, and in particular a neurodegenerative condition. The medical panel which oversees this process should include a senior neurologist, a senior psychiatrist and a senior neuropsychologist, with backup support where the case is complex or critical decisions have to be made. The same requirement could apply to those under the age of 70 years whose performance or behaviour is a cause for concern, but we consider that it should routinely occur for Heads of State. We have a duty to ensure that Heads of State are not compromised in their decision-making and related cognitive skills, as this could result in statements and actions that have a substantive adverse impact on citizens in their own country and in other countries. A positive outcome from an annual medical screen would also be helpful to the person in question, both in terms of providing him/her with reassurance, and also being able to indicate to others that their state of health is not a significant issue.

## Declarations

**Ethical approval:** Ethical approval was not required for this article, since it does not include data relating to clinical research involving patients.

**Guarantor:** NK.

**Contributorship:** NK drew up an initial draft of the paper. SB provided input to this draft, and helped prepare the final version for submission.

**Provenance:** Not commissioned; editorial review.

**Use of generative AI:** No generative AI was used during the preparation of this manuscript.
